# The Clinical Laboratory

**Published:** 1913-02-22

**Authors:** 


### THE CLINICAL LABORATORY.

### III. The Clinical Laboratory in Diabetes.

A good clinical laboratory is absolutely essential to the proper study and treatment of cases of diabetes. A full discussion of the methods used for this purpose would fill a volume, and here it will merely be indicated in what manner the laboratory can help the physician. The collection of the urine is one of the most important matters, for inaccuracy in the necessary details renders useless all careful laboratory work. The essential points are that all the urine be collected, that it be protected from fermentation and from ammoniacal decomposition. Urine passed during defæcation must be estimated by the nurse and allowed for. The simplest manner of collecting the urine is for the successive specimens to be placed immediately in a stoppered Winchester quart which contains about half an ounce of the cheapest chloroform. All the bottles filled are then sent to the laboratory, where they are measured and mixed before the samples are taken for analysis.

What is the best method for estimating sugar? Personally the writer prefers Benedict's (see Emerson's "Diagnosis"), but the great essential is to be familiar with one method and to check one's results by examination of glucose solutions of known strength. Where the titrations are left to an assistant it is absolutely necessary to check his or her results in this manner. One great advantage is that chloroform does not interfere.

The diet of all diabetics should be weighed and carefully controlled in all particulars, not merely as regards carbohydrates. One can often see that the institution of a rigid diet on the old lines is followed by slight, if any, reduction of the glycosuria. It is often followed by coma in a very unexpected way. A good plan is to give the ordinary diet for a few days and estimate the sugar, and then change to a diet which consists solely of half a pound of butter with six to eight ounces of oatmeal. The latter can be given in the form of porridge or oatcakes. This diet almost invariably diminishes the sugar, and will suffice to render many patients aglycosuric. The oatmeal may be still further diminished if the sugar persists. It is usual to precede the oatmeal "cure" by a fast day, but this is not without danger. What is the *raison d'être* of the oatmeal cure? Originally it was advised for the diminution of acidosis, and there is no doubt that in many cases it was successful for a time. It was supposed that the oatmeal had some specific virtues. This is probably incorrect. Its success is probably due to the fact that acidosis is due to inability of the body to obtain (or utilise when obtained) carbohydrates, whilst under the conditions of the "cure" the diabetic can utilise even a fairly large amount of carbohydrate. When a patient on an oatmeal dietary is given additional meat, the sugar rises in the course of a day or two, to fall on a second withdrawal of meat. It appears that animal proteins have some pernicious action on the metabolism of carbohydrates, a good reason for keeping a close watch upon the nitrogen balance in diabetes.

In practically all cases of diabetes there comes a time when the onset of acidosis complicates the picture. The acid intoxication in diabetes is due to $\beta$-oxy-butyric acid and its decomposition product aceto-acetic or di-acetic acid. Whether the phenomena of coma are due to acidity of the blood is still an open question which cannot be discussed here. There can be no doubt but that the process which results in the formation of these acids is the cause of death in many cases of diabetes. That being so, it is very desirable that we should have some laboratory guide which will enable us to gauge the degree of acidosis. The simplest is the long-known "iron reaction" of the urine. To a sample of urine is added solution of perchloride of iron, and the presence of di-acetic acid is revealed by a port-wine coloration. It is often stated that urine which has been boiled does not give this reaction, but this is not true in most cases. Attempts have been made to use this reaction for quantitative work by means of a colorimeter. One can with a little experience form a rough idea from the intensity of the colour. The $\beta$-oxy-butyric acid may be extracted from the urine, but this is a long, tedious, and expensive process. Polarimetry after fermentation also gives a rough idea of the degree of acidosis. It is possible to gain a rough idea of the quantity of organic acid in the urine by means of Folin's method (see Barnes, *British Medical Journal*, January 29, 1910), and to gauge the danger by the ammonia-nitrogen coefficient. The latter method depends on the fact that acids when given in great excess or produced within the body are excreted from the body in combination with ammonia so that ammonia appears in the urine in place of some of the urea. Normally the ammonia-nitrogen of the urine forms about 3 to 5 per cent. of the total nitrogen. The total nitrogen is easily estimated by Kjeldahl's method when the laboratory has been fitted with a proper set of apparatus. The ammonia-nitrogen is easily estimated by the formalin process, which only takes a few minutes. Take some commercial formalin solution, add a drop of phenolphthalein solution, and then dilute caustic-soda solution until the solution has the faintest pink tinge. Then take 20 c.c. urine, dilute with distilled water, add excess of neutral oxalate of potassium crystals, and titrate with decinormal soda solution, using phenolphthalein solution as an indicator. When the urine has reached a definite pink tinge add some of the neutral formalin solution (10 c.c.). The pink tinge disappears at once. Add more soda solution until the colour reappears. Suppose this second part of the titration requires $x$ c.c., then the total nitrogen which is excreted in the form of ammonia per diem $= \frac{x}{20} \times 0.0014 \times A$, where A = number of c.c. in total urine.

When this figure reaches three grams the patient is in great danger of coma, and should certainly be treated by large doses of sodium bicarbonate.

